# Supplementary material for: Compromised DNA repair is responsible for diabetes‐associated fibrosis
Source: EMBO J. 2020 Apr 27;39(11):e103477. doi: 10.15252/embj.2019103477 (PMC7265245; doi:10.15252/embj.2019103477)
Supplement: Supplementary file 1 — Appendix [file EMBJ-39-e103477-s001.pdf]

Compromised DNA repair is responsible for diabetes-associated organ fibrosis

Kumar Varun, Agrawal Raman, Pandey Aparamita, Kopf Stefan, Hoeffgen Manuel, Kaymak Serap, Bandapalli Obul Reddy, Gorbunova Vera, Seluanov Andrei, Mall Marcus A, Herzig Stephan, Nawroth Peter P

Appendix Figures

- Appendix Figure S1 (A-B)
- Appendix Figure S2 (A-D)
- Appendix Figure S3 (A-D)
- Appendix Figure S4 (A-D)
- Appendix Figure S5 (A-D)
- Appendix Figure S6 (A-B)
- Appendix Figure S7 (A-E)
- Appendix Figure S8 (A-C)
- Appendix Figure S9 (A-B)
- Appendix Figure S10
- Appendix Figure S11 (A-F)

Appendix Tables

- Appendix Table S1
- Appendix Table S2
- Appendix Table S3
- Appendix Table S4
- Appendix Table S5

Appendix Figure S1:

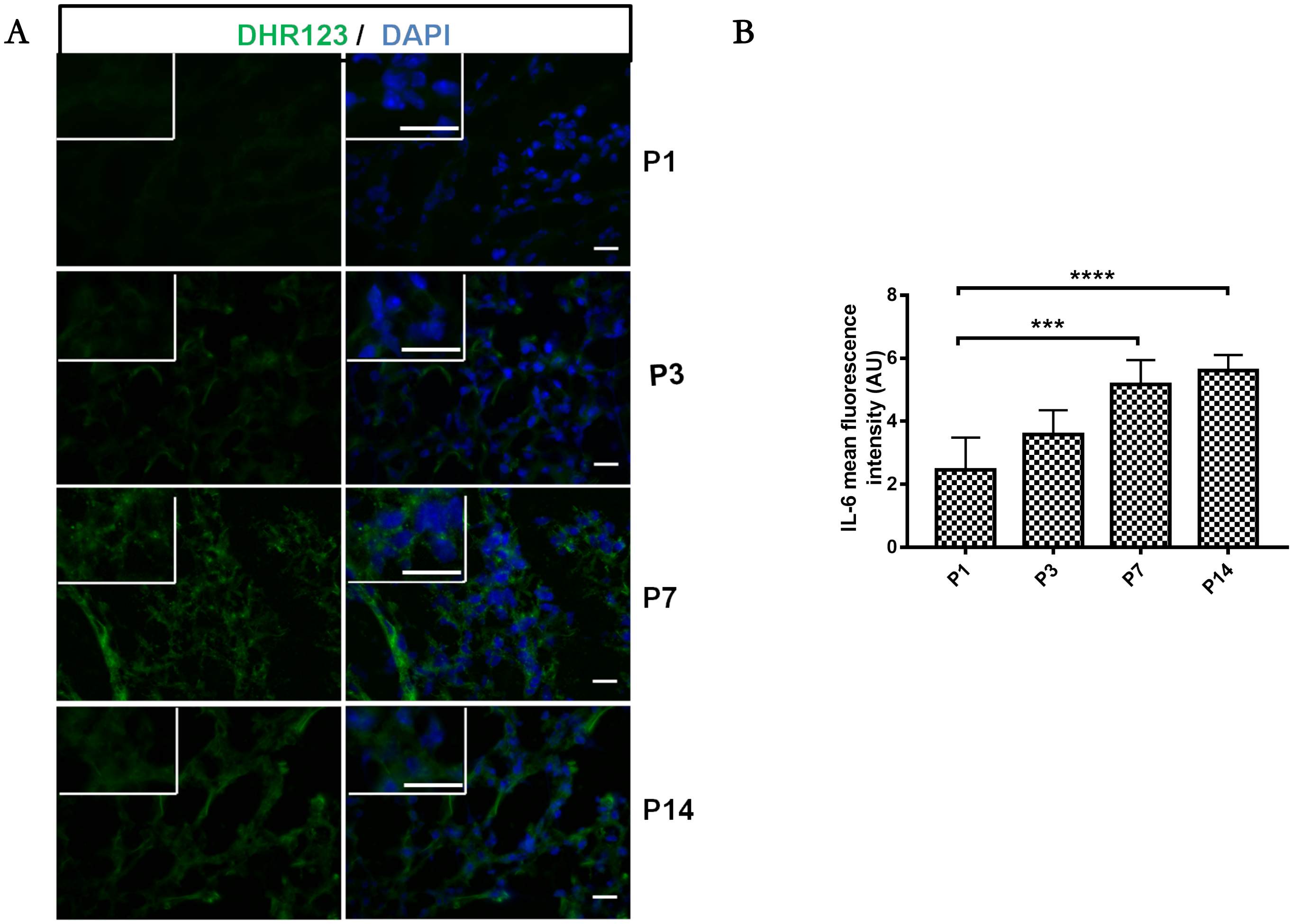

Legend Appendix Figure S1

(A) Representative images of lungs from postnatal pups at the indicated age (in days), stained for the peroxide linked oxidative stress marker DHR-123. The lungs studied in this Figure and in Figure 1c are identical (Scale 10μm).

(B) Mean fluorescence intensity of IL-6 positive cells in lungs from postnatal pups at the indicated age (in days), as determined by immunofluorescence analysis (mean ± SD, *** $p<0.001$, **** $p<0.0001$, N=8).

Appendix Figure S2:

A

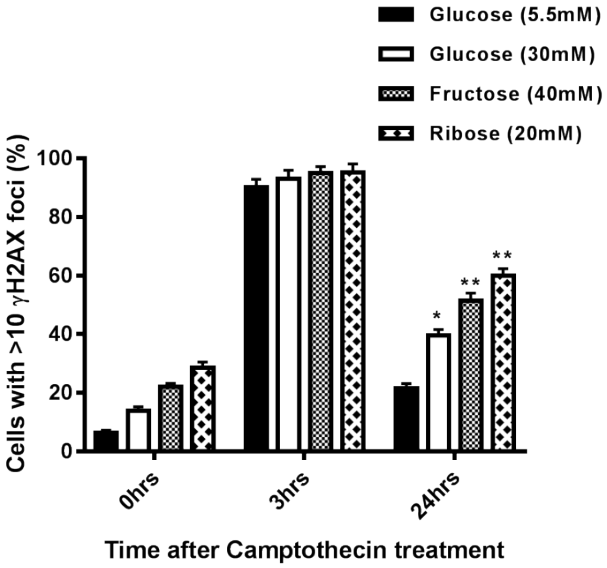

B

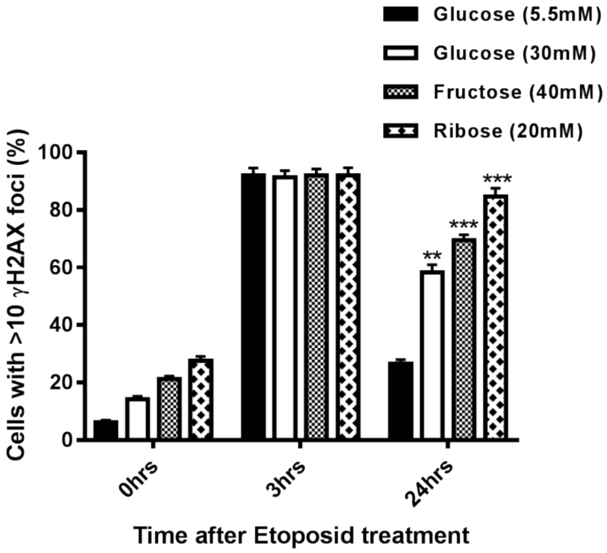

C

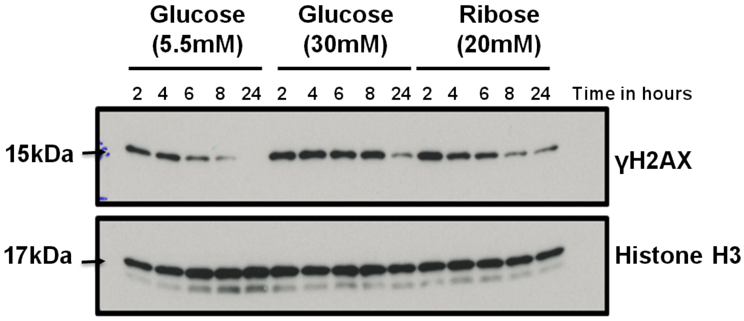

D

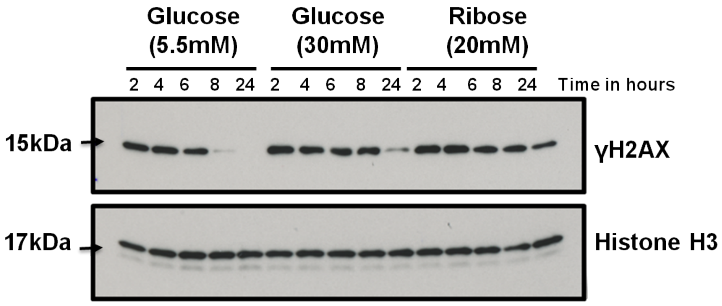

Legend Appendix Figure S2:

(A) Mean percentage of DSB positive nuclei in camptothecin treated (1μM) murine lung fibroblasts as evidenced by γH2AX positivity after culture in the presence of different reducing carbohydrates at 0, 3 or 24 hours. More than 400 cells were analyzed for each bar (mean ± SD, *: $p<0.05$, **: $p<0.01$).

(B) Mean percentage of DSB positive nuclei in etoposide treated (5μM) murine lung fibroblasts as evidenced by γH2AX positivity after culture in the presence of different reducing carbohydrates at 0, 3 or 24 hours. More than 400 cells were analyzed for each bar (mean ± SD, **: $p<0.01$, ***: $p<0.001$).

(C) Representative immunoblots from lysates of HEK293 cells cultured in the presence of the reducing sugars as indicated and treated with etoposide (5μM) were probed for the DNA damage marker γH2AX. Histone-H3 used as a loading control.

(D) Representative immunoblots from the lysates of murine podocytes, cultured in the presence of reducing sugars indicated, then treated with etoposide (5μM) and probed for the DNA damage marker γH2AX. Histone-H3 used as a loading control.

Appendix Figure S3:

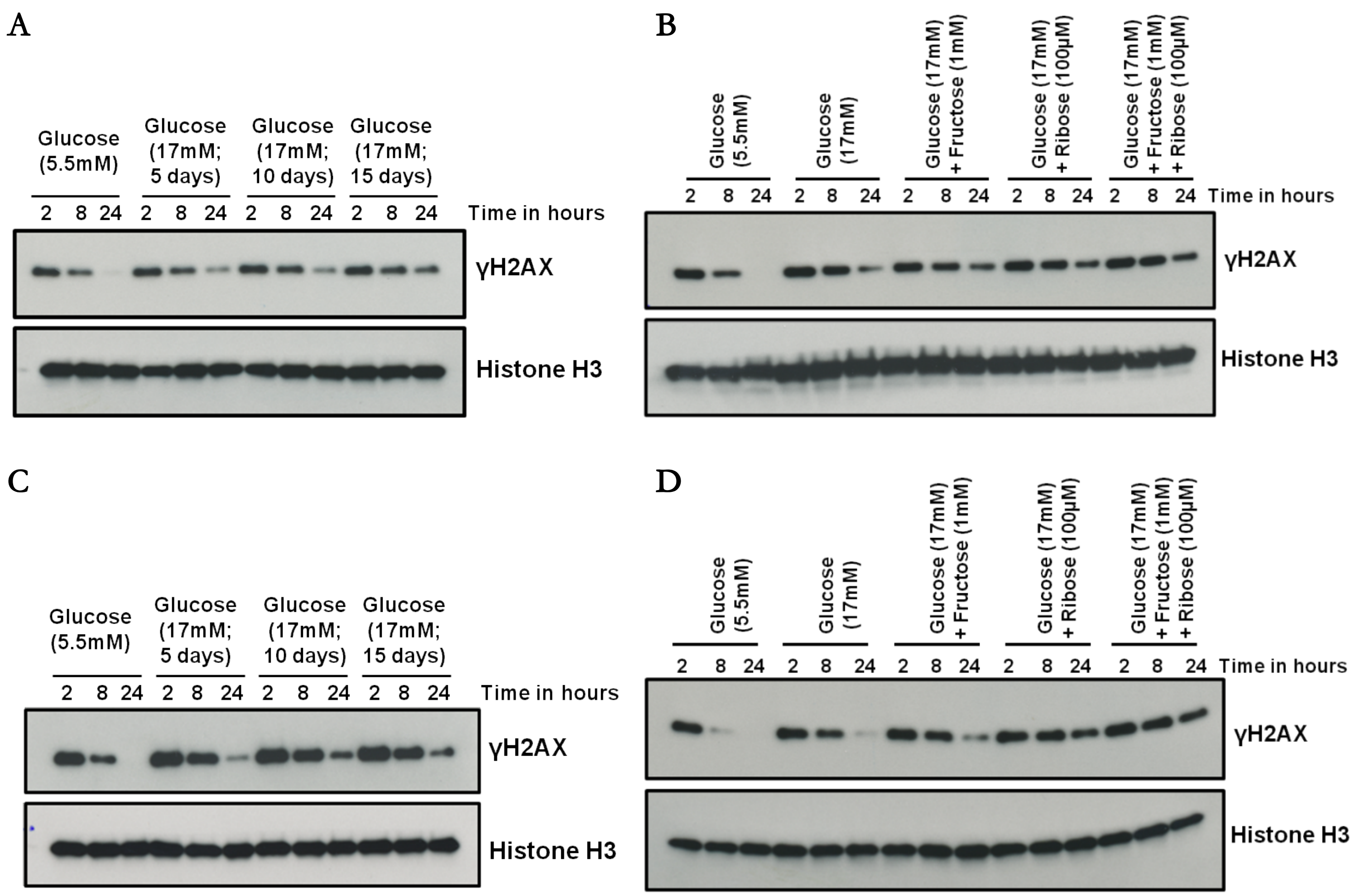

Legend Appendix Figure S3

(A) Representative immunoblots from lysates of A549 cells cultured in the presence of the indicated glucose concentrations for 5, 10 or 15 days and treated with etoposide (5μM; upper panel) were probed for the DNA-DSBs marker γH2AX. Histone-H3 used as a loading control.

(B) Representative immunoblots from lysates of A549 cells cultured in the presence of the indicated glucose concentrations along with or without fructose and/or ribose for 5 days and treated with etoposide (5μM; upper panel) were probed for the DNA-DSBs marker γH2AX after the indicated intervals. Histone-H3 used as loading control.

(C) Representative immunoblots from lysates of HEK-293 cells cultured in the presence of the indicated glucose concentrations for 5, 10 or 15 days and treated with etoposide (5μM; upper panel) were probed for the DNA-DSBs marker γH2AX. Histone-H3 used as a loading control.

(D) Representative immunoblots from lysates of HEK-293 cells cultured in the presence of the indicated glucose concentrations along with or without fructose and/or ribose for 5 days and treated with etoposide (5μM; upper panel) were probed for the DNA-DSBs marker γH2AX after the indicated intervals. Histone-H3 used as a loading control.

Appendix Figure S4:

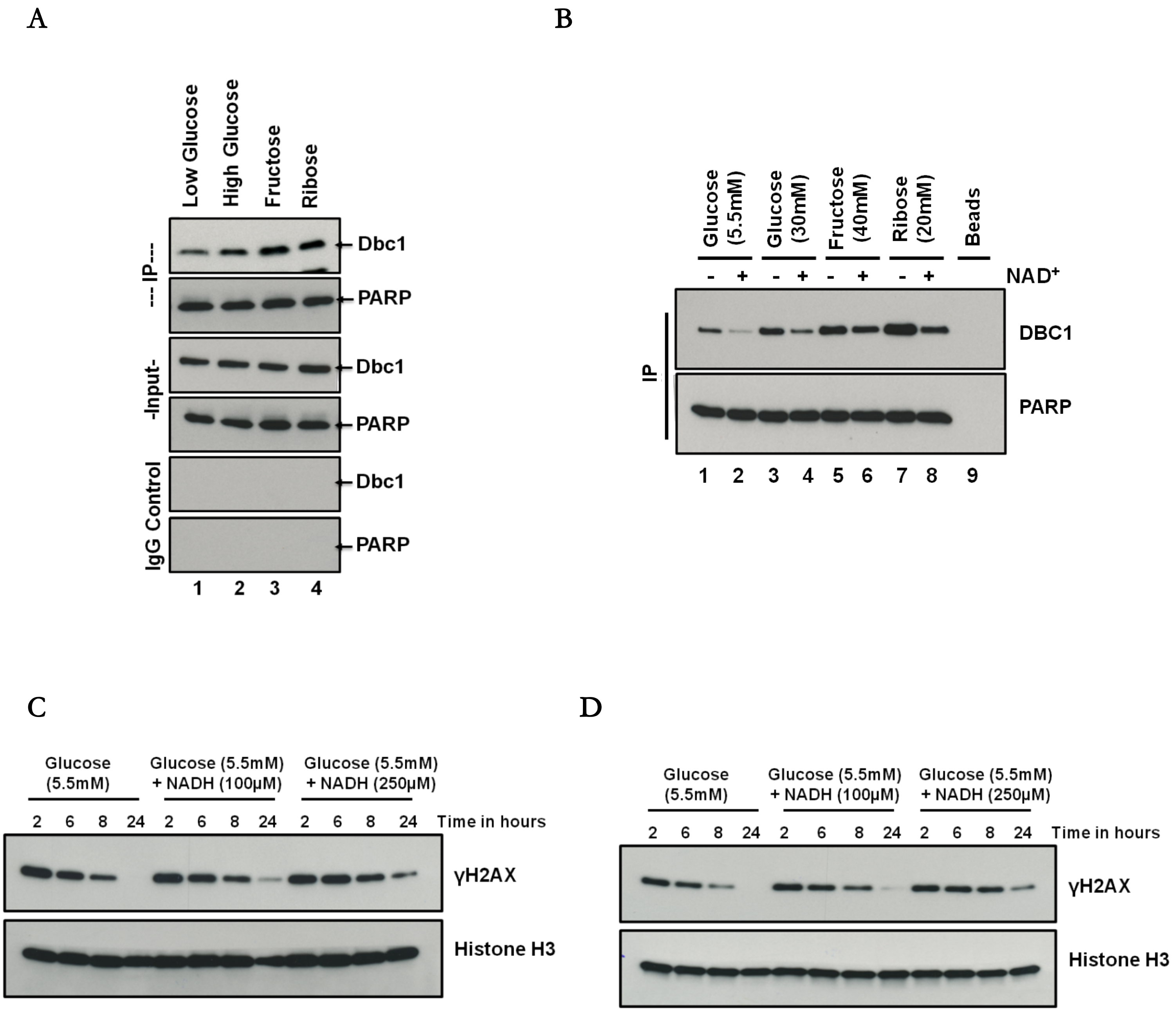

Legend Appendix Figure S4:

(A) Primary murine lung fibroblasts were cultured in either low (5.5mM; lane 1) or high glucose (30mM, lane 2) for 5 days or with fructose (40mM; lane 3) or ribose (20mM; lane 4) for 3 days. The cell extract was immunoprecipitated using anti-PARP, or a non-specific species control antibody (lane 4 and 9) PARP, or its interacting partner DBC-1, was detected using a PARP or a DBC-1 specific antibody. The incubation time with fructose and ribose had to be reduced to 3 days only due to toxicity.

(B) A549 cells cultured in either low (5.5mM) or high glucose (30mM) for 5 days, or with fructose (40mM), or ribose (20mM) for 3 days (graph bar 4). The cell extract was immunoprecipitated using anti-PARP with, or without the presence of NAD+ (500µM) in the wash buffer or a non-specific species control antibody (lane 4 and 9). PARP or its interacting partner DBC-1 was then detected using a PARP, or DBC-1 specific antibody. The experiments presented in this Figure and in Figure 2F were performed simultaneously. The incubation time with fructose and ribose had to be reduced to 3 days only due to toxicity.

(C) Representative immunoblots from lysates of A549 cells growing under normal culture conditions and either left untreated or treated with NADH (100µM or 250µM) for 24 hours. The DNA-DSBs were induced by etoposide (5µM; upper panel), and their ability to repair the damaged DNA was monitored, for the indicated time points by probing DNA damage marker γH2AX. Histone H3 used as a loading control.

(D) Representative immunoblots from lysates of HEK-293 cells growing under normal culture conditions and either left untreated or treated with NADH (100µM or 250µM) for 24 hours. The DNA-DSBs were induced by etoposide (5µM; upper panel), and their ability to repair the damaged DNA was monitored, for the indicated time points by probing DNA damage marker γH2AX. Histone H3 used as a loading control.

Appendix Figure S5:

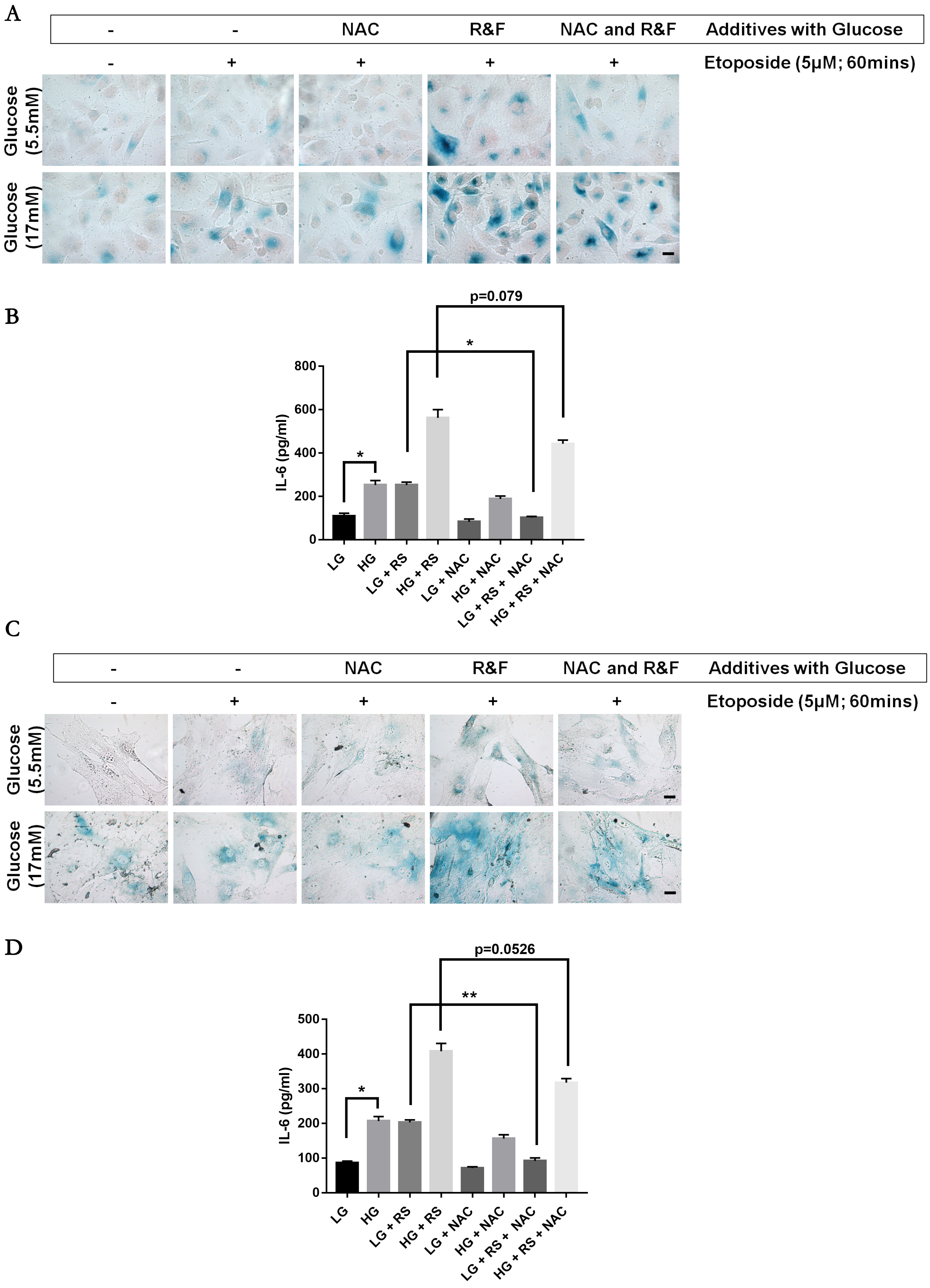

Legend Appendix Figure S5

(A) Representative images of A549 cells cultured in the presence of the indicated glucose concentrations for 5 days alone or with other reducing sugars (here F indicates for fructose at concentration 1mM, and R indicates for Ribose at concentration 100µM) along with NAC (N-Acetyl cysteine at 2mM) and treated with etoposide (5µM for 60 minutes), washed and cultivated in the sugars as indicated for 10days and stained for β-galactosidase, as indicated in methods. Eosin used as a morphology stain (Scale 40µm).

(B) Quantitative analysis of the IL-6 secreted in the culture supernatant of A549 cells treated as described in appendix figure S5A. (mean ± SD, *: $p<0.05$).

(C) Representative images of murine podocytes cultured in the presence of the indicated glucose concentrations for 5 days alone or with other reducing sugars (here F indicates for fructose at concentration 1mM, and R indicates for Ribose at concentration 100µM) along with NAC (N-Acetyl cysteine at 2mM) and treated with etoposide (5µM for 60 minutes), washed and cultivated in the sugars as indicated for 10days and stained for β-galactosidase as indicated in methods. Eosin used as a morphology stain (Scale 40µm).

(D) Quantitative analysis of the IL-6 secreted in the culture supernatant of murine podocytes treated as described in appendix figure S5C. (mean ± SD, *: $p<0.05$, **: $p<0.01$).

Appendix Figure S6:

A

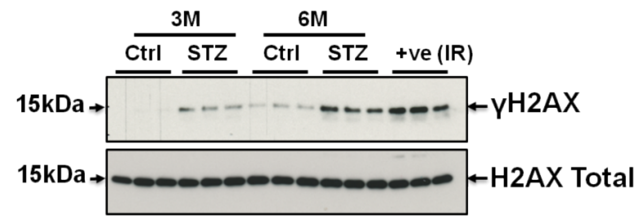

B

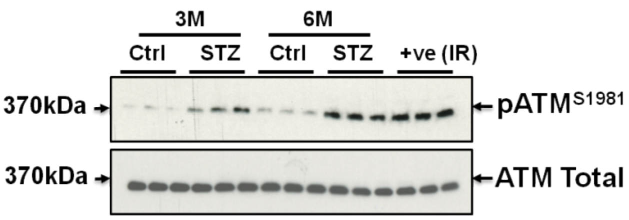

Legend Appendix Figure S6:

(A) Representative immunoblots of lungs harvested from 3- or 6- months non-diabetic control or STZ diabetic mice and probed for γH2AX. Total-H2AX used as loading control.

(B) Representative immunoblots of lungs harvested from 3- or 6- months non-diabetic control or STZ diabetic mice and probed for the DNA damage sensor kinase pATM. Total ATM used as a loading control.

Appendix Figure S7:

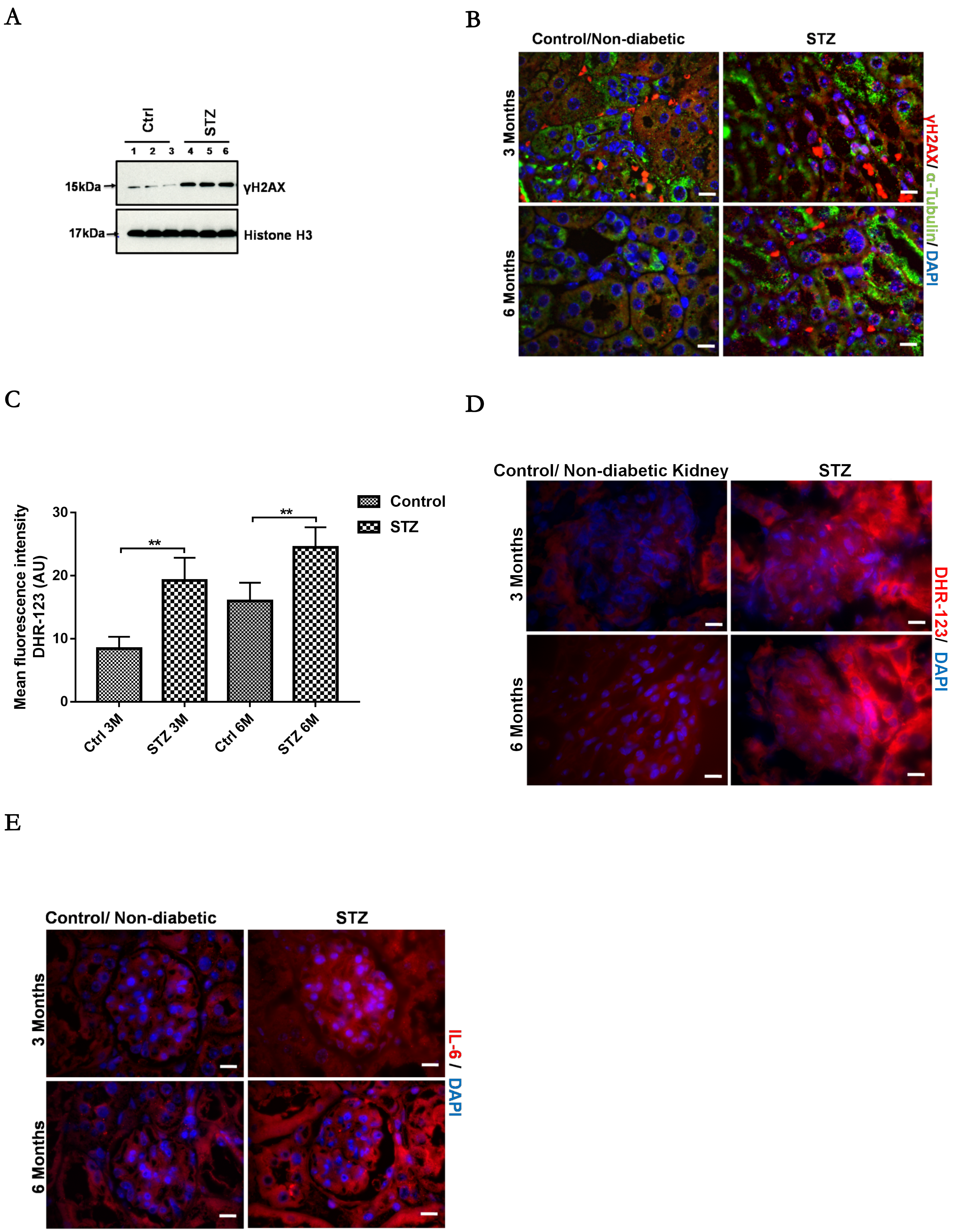

Legend Appendix Figure S7:

(A) Representative immunoblots of kidneys harvested from 6- months non-diabetic control or STZ-diabetic mice and probed for γH2AX. Histone-H3 used as a loading control.

(B) Representative images of kidneys for nuclei positive for the DNA-DSB marker γH2AX, versus DAPI, in age-matched 3-, or 6- months control, versus STZ induced diabetic mice, as determined by immunofluorescence analysis (scale 10μm).

(C) Quantitative analysis of the peroxide linked oxidative stress marker DHR-123 in age-matched 3-, or 6- month control lungs, or lungs from STZ-diabetic mice. DHR-123 was determined by mean fluorescence intensity of respective lungs.

(D) Representative images of kidneys from age-matched control versus 3- or 6-month STZ-diabetic mice, stainedfor the peroxide linked oxidative stress marker DHR-123, as determined by mean fluorescence intensity of respective kidneys stained with DHR-123 in red. Blue nuclear staining represents DAPI (Scale 10μm).

(E) Representative images of kidneys from age-matched control versus 3- or 6-month STZ-diabetic mice, stained for the SASP marker IL-6, as determined by mean fluorescence intensity of respective kidneys stained with anti-IL-6 in red. Blue nuclear staining represents DAPI (Scale 10μm).

Appendix Figure S8:

A

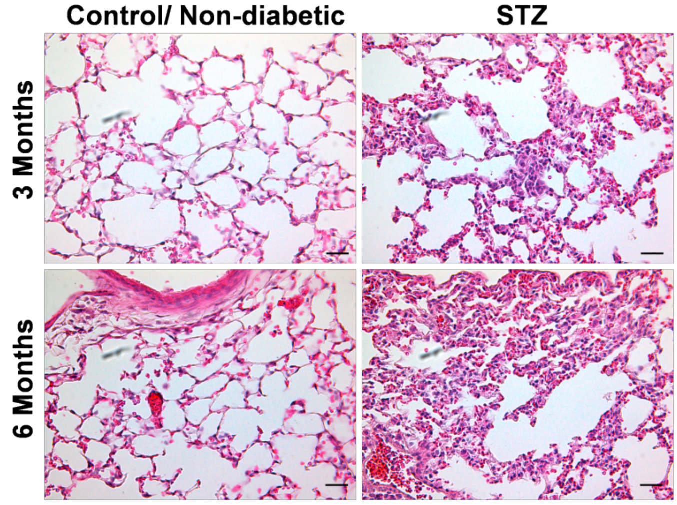

B

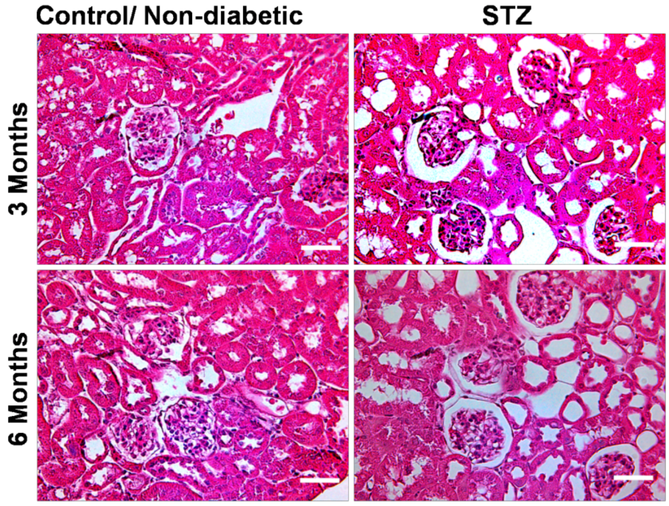

C

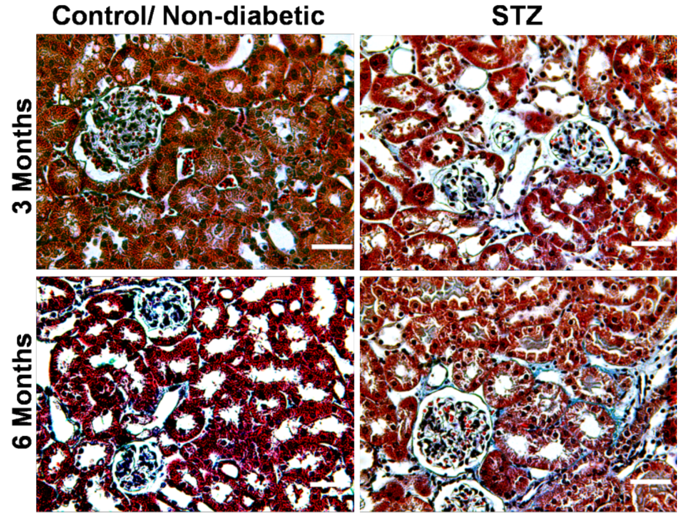

Legend Appendix Figure S8:

(A) Representative images of H&E stained lung sections from age-matched 3- or 6- months control, or STZ-induced diabetic mice, as described in Methods. H&E staining was visualized by bright field and polarized light (Scale 40µm).

(B) Representative images of H&E stained kidney sections from age-matched 3- or 6- months control, or STZ induced diabetic mice, as described in Methods. H&E staining was visualized by bright field and polarized light (Scale 40µm).

(C) Representative images of kidneys from age-matched 3- or 6-month control versus STZ-induced diabetic mice, stained for Masson's Trichrome stain, as described in Methods and visualized by bright field and polarized light, ECM is recognized by its blue staining (Scale 40µm).

Appendix Figure S9:

A

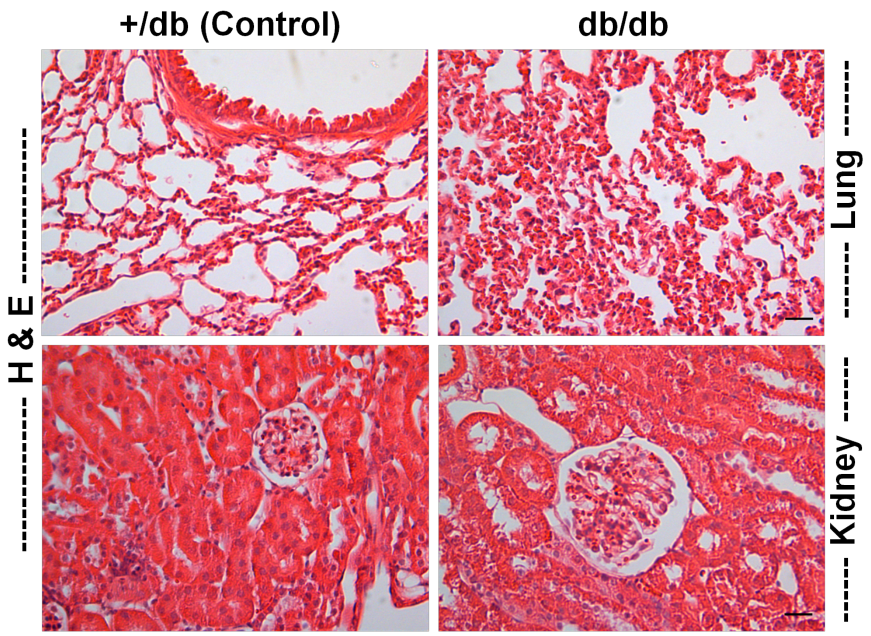

B

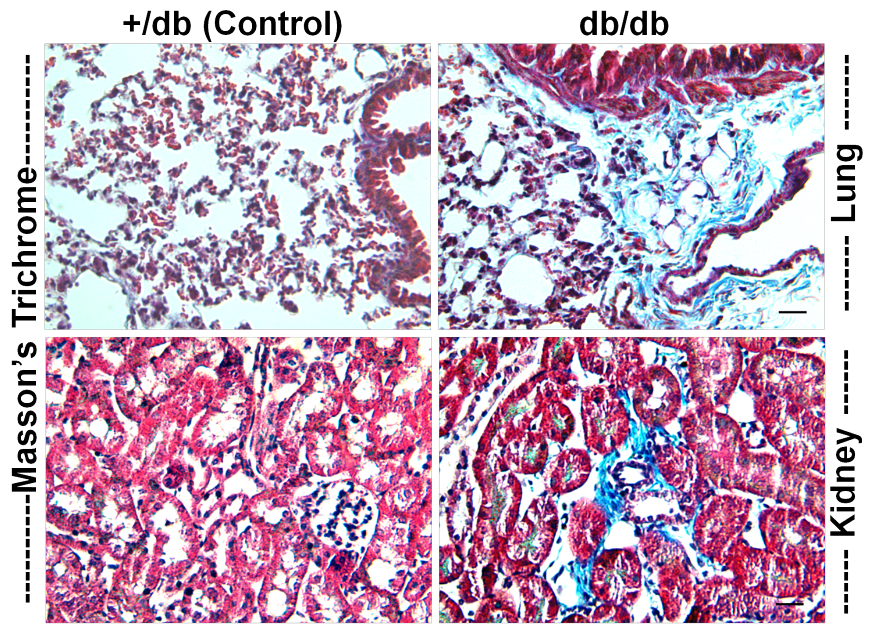

Legend Appendix Figure S9:

(A) Representative images of H&E stained lung or kidney sections from 4-months old lean controls (+/*db*), or age-matched *db/db* diabetic mice, as described in Methods. H&E staining was visualized by bright field and polarized light (Scale 40μm).

(B) Representative images of lungs and kidneys from 4-months old lean controls (+/*db*), or age-matched *db/db* diabetic mice stained with Masson's Trichrome stain, as described in Methods and visualized by bright field and polarized light, ECM is recognized by its blue staining (Scale 40μm).

Appendix Figure S10:

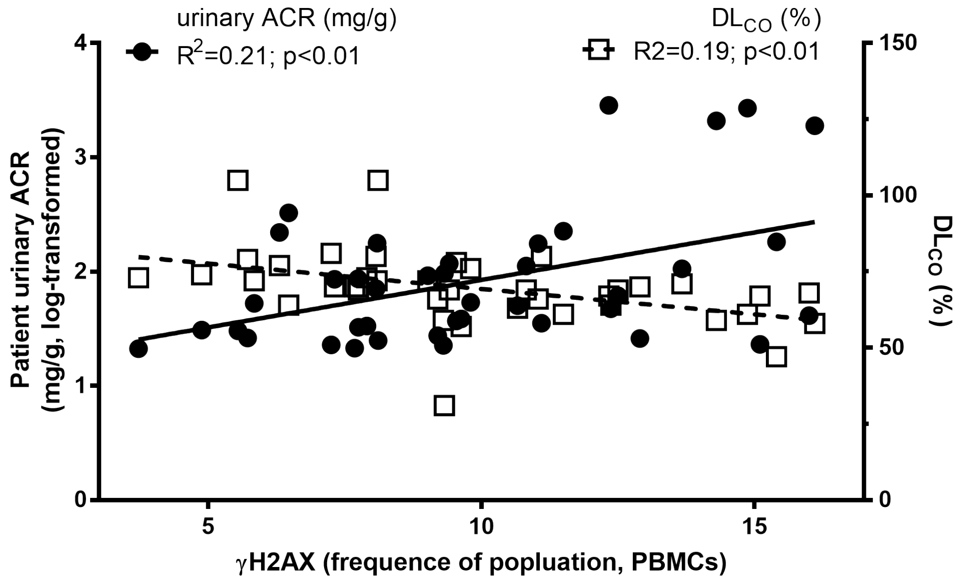

Legend Appendix Figure S10:

Correlation between γH2AX with clinical scores of urinary albumin-creatinine-ratio (ACR) and diffusion capacity (DLco). Urinary ACR is given on the right y-axis, DLco is given on the left y-axis. Urinary ACR was log-transformed to achieve normal distribution. Correlation analysis was calculated via Spearman rho and R2-values and p-values were given in the figure.

Appendix Figure S11:

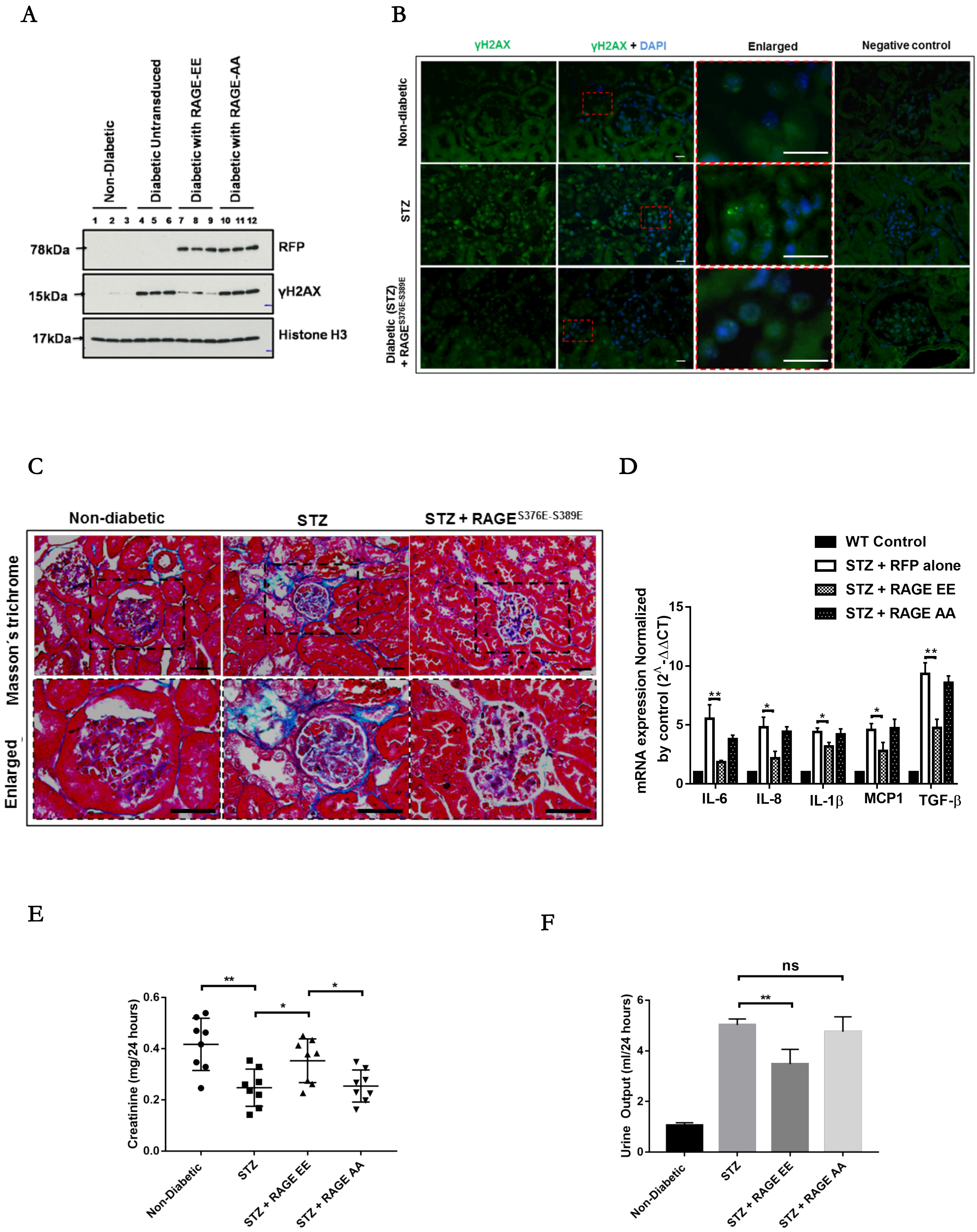

Legend Appendix Figure S11:

(A) Representative immunoblots showing the expression of RFP-RAGE (AA or EE) and γH2AX in transduced kidneys of diabetic mice. Data presented in this Figure, as well as in Figure 5 and EV Figure 5, are from the same mice group.

(B) Representative images of nuclei positive for the DNA-DSB marker γH2AX in kidneys from 6-month STZ-diabetic mice and control mice, transduced with the indicated AAV2/8 virions as described in Methods. The kidneys were harvested 6-weeks after viral transduction. No primary antibody control served as a negative control of the staining. Mean ± SD of 6 animals per group is shown (Scale 10μm).

(C) Representative images of Masson's Trichrome stain for respective RAGE virions transduced kidneys as described in Methods and visualized by bright field and polarized light, where the accumulated ECM or senescent areas are recognized by its blue staining (Scale 40μm). The dotted lines represent the respective zoomed window.

(D) Quantitative analysis of transduction effects of RAGE (AA or EE) or RFP on pro-inflammatory, fibrotic and SASP gene expression in kidney tissue. The mRNA of pro-inflammatory, fibrotic and SASP cytokines were significantly suppressed in RAGE-EE transduction group. The data was normalized against control value (mean ± SD, *: $p<0.05$, **: $p<0.01$).

(E) Quantitative analysis of the urinary creatinine in urine from 6-month STZ-induced diabetic mice, transduced with AAV2/8 as described in Figure EV5A (mean ± SD, *: $p<0.05$, **: $p<0.01$, N=8). Data presented in this Figure, as well as in Figure 5 and EV 5, are from the same mice group.

(F) Quantitative analysis of the total urinary volume excreted in 24 hours from 6-month STZ induced diabetic mice, transduced with AAV2/8 as described in Figure EV5A (mean ± SD, **: $p<0.01$, N=8). Data presented in this Figure, as well as in Figure 5 and EV Figure 5, are from the same mice group.

Appendix Tables:

Appendix table S1: Substances used for DNA damage induction

Drug	Purpose	Concentration	References
Camptothecin	DNA DSB induction	1μM (for 60mins)	(Polo, Blackford et al. 2012)
Etoposide	DNA DSB induction	5μM (for 60mins)	(Dobbin, Madabhushi et al. 2013)

Appendix table S2: Basic characterization of diabetic patient cohort$

Parameters	Controls	Type 1 Diabetes	Type 2 Diabetes	p-value
**Number**	n=44	n=35	n=110	
**Age (years)**	54.3 ± 10.9	51.2 ± 18.5	62.2 ± 9.5	<0.01
**Gender (f/m)**	31/13	15/20	50/60	<0.05
**BMI (kg/m2)**	27.1 ± 4.8	26.1 ± 4.1	32.3 ± 6.4	<0.01
**Diabetes duration (years)**	----	24.8 ± 16.7	11.9 ± 10.3	<0.001
**CVD (yes, n [%])**	1 [2]	3 [9]	8 [7]	n.s.
**Neuropathy (yes, n [%])**	0 [-]	15 [43]	57 [52]	<0.001
**Retinopathy (yes, n [%])**	0 [-]	9 [26]	25 [23]	<0.01
**Nephropathy (yes, n [%])**	0 [-]	6 [17]	35 [32]	<0.01
**Fasting glucose (mg/dl)**	92.0 ± 8.4	197.0 ± 70.0	151.5 ± 44.5	<0.01
**HbA1c (%)**	5.4 ± 0.3	8.2 ± 1.5	7.2 ± 1.3	<0.001
**Triglyceride (mg/dl)**	95.8 ± 33.3	119.8 ± 83.0	180.8 ± 102.2	<0.01
**Cholesterol (mg/dl)**	207.3 ± 41.3	203.2 ± 44.4	189.0 ± 45.4	<0.05
**eGFR (ml/min*1.73m2)**	92.1 ± 13.3	97.6 ± 23.2	97.2 ± 26.8	n.s.
**urinary ACR (mg/g)***	6.7 [4.7/11.5]	6.6 [4.7/15.0]	10.7 [5.7/30.7]	<0.05

$:Data given in mean ± SD, or n[%], or median [25./75. percentile]†

Group differences were calculated via one-way-ANOVA or X^2-Test

Appendix table S3: Details of plasmids used for AAV production

**Packaged AAVs**	**Plasmid**
RAGE RFP $^{S376E-S389E}$	pAM CBA RAGE RFP (EE) WPRE bGH& pDP2rs + AAV8.
RAGE RFP $^{S376A-S389A}$	pAM CBA RAGE (AA) RFP WPRE bGH& pDP2rs + AAV8.
RFP alone	pAM CBA RFP WPRE bGH& pDP2rs + AAV8.

Appendix table S4: Details of primary and secondary antibodies

Antibody	Species	Supplier	Application
CD11-b	Mice	R & D	IF
DBC1	Mice	5857(Cell Signaling)	IB, IP
FLAG	Rat	MA1-142-A647 (Thermo)	IF/FC
HDAC1	Rabbit	2062(Cell Signaling)	IB
Histone H1	Mouse	(Abcam)	IB
Hist-H3 S10	Rabbit	9701(Cell Signaling)	IB, IF
ɤH2AX	Rabbit	9718(Cell Signaling)	IB, IF
ɤH2AX	Mouse	05-636 (Millipore)	IF
IL-6	Rabbit	Ab6672(Abcam)	IF
pATM	Mouse	201-300-400(Rockland)	IF, IB
pSQE	Rabbit	9607(Cell Signaling)	IB
PARP	Goat	(R&D)	IB
PARP	Mice	NB (Novus Biologicals)	IF

RAGE	Goat	Sc-8230(Santa Cruz)	IF, IP, IB
RAGE	Rat	MAB1179(R&D)	IF
Tubulin	Rabbit	(Sigma)	IF/IB
53BP1	Rabbit	NB100-304 (Novus Biologicals)	IF
8-OHdG	Mice	AM03160PU-N (Acris)	IF
**Secondary antibodies**			
Anti-Goat-HRP	Donkey	sc-2033 (Santa Cruz)	IB
anti-Mice IgG-HRP	Goat	sc-2062 (Santa Cruz)	IB
anti-Rabbit IgG-HRP	Goat	7074(Cell Signaling)	IB
anti-Mice IgG-HRP	Goat	7076 (Cell Signaling)	IB
anti Goat Alexa-647	Donkey	Ab150139 (Abcam)	IF

Anti Rabbit-Alexa-555	Donkey	Ab150074 (Abcam)	IF
Anti-Mice Alexa-647	Donkey	Ab150111 (Abcam)	IF
Anti Rat-Alexa-647	Donkey	Ab150155 (Abcam)	IF

Appendix table S5: Cloning primer details

	Name	Sequence
1	RAGE S376A F	GAAGGCCCCGGAAGCCCAGGAGGATGAG
2	RAGE S376A R	ATCCTCCTGGGCTTCCGGGGCCTTCCTC
3	RAGE S390A F	GTGCAGAGCTGAATCAGGCGGAGGAAGCGGAGATG
4	RAGE S390A R	CATCTCCGCTTCCTCCGCCTGATTCAGCTCTGCAC
5	RAGE S376E F	GAAGGCCCCGGAAGAACAGGAGGATGAG
6	RAGE S376E R	CTCATCCTCCTGTTCTTCCGGGGCCTTC
7	RAGE S390E F	GTGCAGAGCTGAATCAGGAAGAGGAAGCGGAGATG
8	RAGE S390E R	CATCTCCGCTTCCTCTTCCTGATTCAGCTCTGCAC
9	Murine mtDNA F	CCCATTCCACTTCTGATTACC
10	Murine mtDNA R	ATGATAGTAGAGTTGAGTAGCG
11	Murine ncDNA F	GTACCCACCTGTCGTCC

12	Murine ncDNA R	GTCCACGAGACCAATGACTG
13	IL-6 F	CAGAAGGAGTGGCTAAGGACC
14	IL-6 R	CGCACTAGGTTTGCCGAGTAG
15	IL-8 F	AGGGAGCCTCCTGTCACTG
16	IL-8 R	AGCCATTCAAGCAGGAAGACC
17	TGF B F	TGGAGCAACATGTGGAACTC
18	TGF B F	CGTCAAAAGACAGCCACTCA
19	IL-1 B F	GAGTGTGGATCCCAAGCAAT
20	IL-1 B F	GGGGAACTCTGCAGACTCAA
25	MCP1 F	TGCATCTGCCCTAAGGTCTTC
26	MCP1 R	AGTGCTTGAGGTGGTTGTGGA
27	GAPDH F	GGGTGTGAACCACGAGAAAT
28	GAPDH R	CCTTCCACAATGCCAAAGTT
