# Supplementary material for: Compromised DNA repair is responsible for diabetes‐associated fibrosis
Source: EMBO J. 2020 Apr 27;39(11):e103477. doi: 10.15252/embj.2019103477 (PMC7265245; doi:10.15252/embj.2019103477)
Supplement: Supplementary file 2 — Expanded View Figures PDF [file EMBJ-39-e103477-s002.pdf]

Expanded View Figures

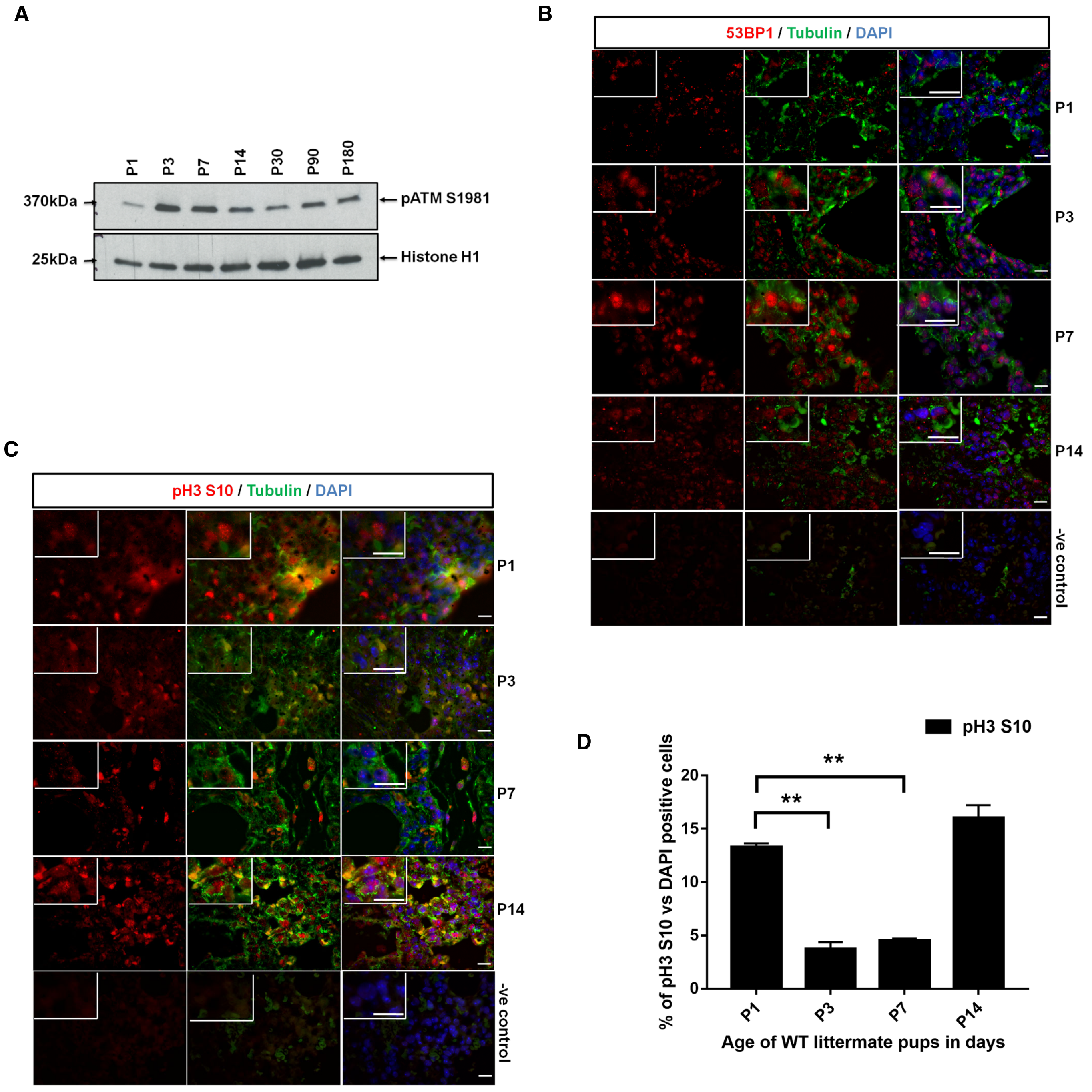

Figure EV1.

Figure EV1. DNA double-strand breaks signaling in mice pups at indicated postnatal days of birth.

A Western blot analysis of pATM-S1981 indicating activation of DDR in postnatal lungs. Histone H1 was used as a loading control.

B Representative immunofluorescence images of postnatal lungs stained for the DNA damage marker 53BP1 (red) at the indicated days. Green cytoplasmic staining represents α-tubulin; blue nuclear staining represents DAPI (scale 10 μm). No primary antibody control served as a negative control (shown P14) of the staining.

C Representative immunofluorescence images of postnatal lungs, stained for the proliferation marker phosphorylated-H3 Ser10 (pH3-S10; red) at the indicated days. Green cytoplasmic staining represents α-tubulin; blue nuclear staining represents DAPI (scale 10 μm). No primary antibody control served as a negative control (shown P14) of the staining.

D Mean percentage of the proliferation marker pH3S10-positive nuclei in postnatal murine lungs, as determined by immunofluorescence analysis (mean ± SD, $^{**}P < 0.01$, $N = 8$).

Source data are available online for this figure.

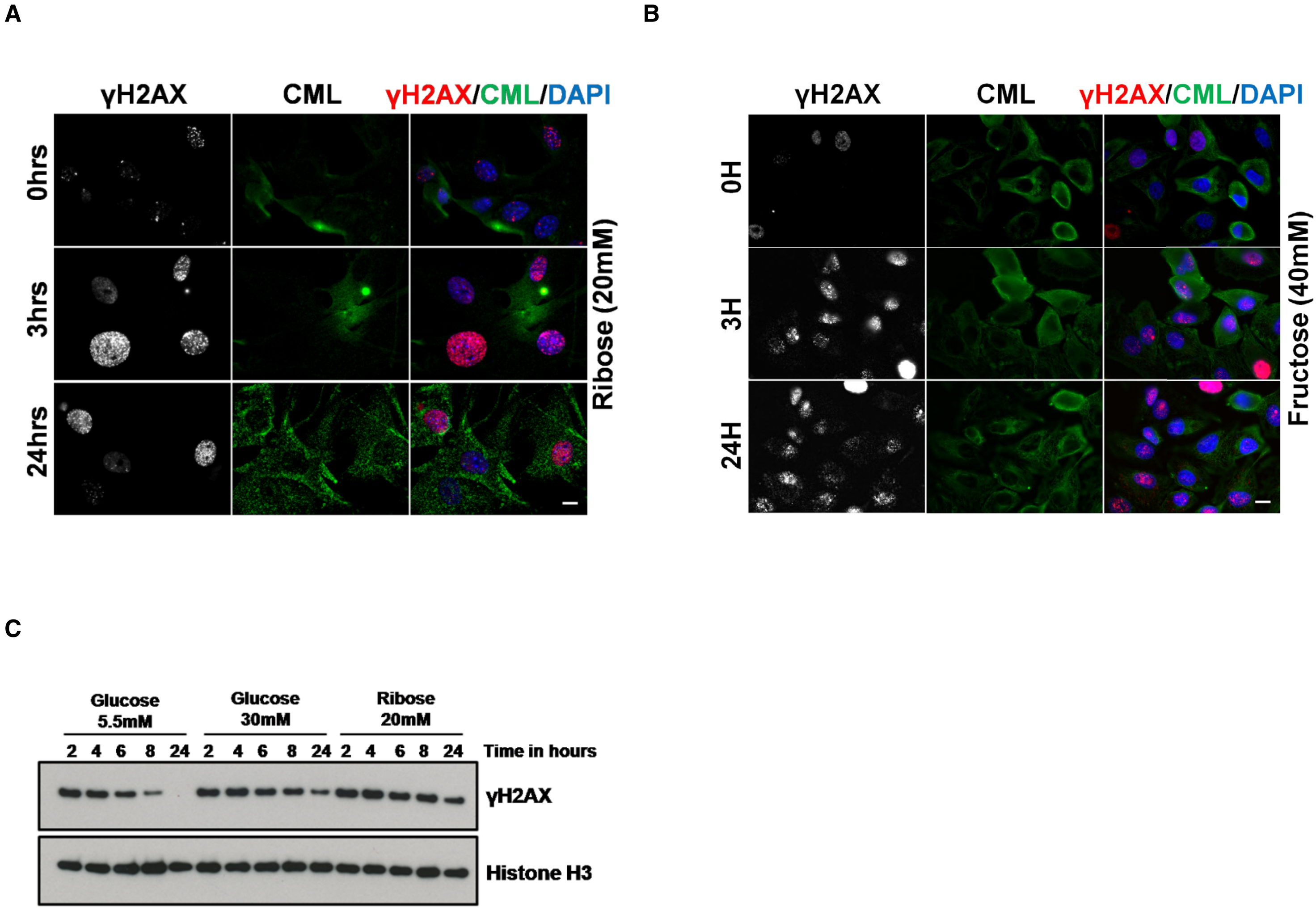

Figure EV2. Molecular association of cellular DNA damage signaling to reducing sugars.

A Immunofluorescence analysis of DSBs-associated foci, marked by γH2AX in lung adenocarcinoma (A549) cells, cultured in ribose (20 mM) for 3 days, and treated with etoposide (5 μM for 60 min). The resolution of DNA-DSBs foci, as marked by γH2AX was monitored over 24 h after drug treatment (scale 10 μm). CML (marked in green) served as induction control. This experiment was performed simultaneously with the data shown in Fig 2A.

B Immunofluorescence analysis of DSBs-associated foci, marked by γH2AX in lung adenocarcinoma (A549) cells, cultured in fructose (40 mM) for 3 days and treated with etoposide (5 μM for 60 min). The resolution of DNA-DSBs foci, as marked by γH2AX, was monitored over 24 h after drug treatment (scale 10 μm). CML (marked in green) served as induction control. This experiment was performed simultaneously with the data shown in Figs 2A and EV3A.

C Representative immunoblots from lysates of A549 cells cultured in the presence of the reducing sugars as indicated and treated with etoposide (5 μM; upper panel) were probed for the DNA damage marker γH2AX. Histone H3 was used as a loading control.

 © 2020 The Authors

Figure EV3. Reducing sugar associated DNA repair defects are causatively linked to sugar stimulation.

A Representative immunoblots from the lysates of A549 cells, cultured in the presence of reducing sugars as indicated, then treated with camptothecin (1 µM; lower panel), and probed for the DNA damage marker γH2AX. Histone H3 used as a loading control.

B Immunofluorescence analysis of DSBs-associated foci, marked by γH2AX, in lung adenocarcinoma (A549) cells, cultured in low glucose (5.5 mM) for 5 days, and then treated with camptothecin (1 µM for 60 min). The resolution of DNA-DSBs foci, as marked by γH2AX, was monitored over 24 h after drug treatment. CML (marked in green) served as induction control (scale 10 µm).

C Immunofluorescence analysis of DSBs-associated foci, marked by γH2AX in lung adenocarcinoma (A549) cells, cultured in high glucose (30 mM) for 5 days, and then treated with camptothecin (1 µM for 60 min). The resolution of DNA-DSBs foci, as marked by γH2AX, was monitored over 24 h after drug treatment. CML (marked in green) served as induction control (scale 10 µm).

D Immunofluorescence analysis of DSBs-associated foci, marked by γH2AX in lung adenocarcinoma (A549) cells, cultured in ribose containing medium (20 mM) for 3 days, and then treated with camptothecin (1 µM for 60 min). The resolution of DNA-DSBs foci, marked by γH2AX, was monitored over 24 h after drug treatment. CML (marked in green) served as induction control (scale 10 µm).

E Immunofluorescence analysis of DSBs-associated foci, marked by γH2AX in lung adenocarcinoma (A549) cells, cultured in fructose containing medium (40 mM) for 3 days, and then treated with camptothecin (1 µM for 60 min). The resolution of DNA-DSBs foci, marked by γH2AX, was monitored over 24 h after drug treatment. CML (marked in green) served as induction control (scale 10 µm).

F Mean percentage of DSBs-positive nuclei, marked by γH2AX, from the immunofluorescence analysis described in Appendix Fig S3D–F (more than 400 cells were analyzed for each bar). Cells cultured in glucose, fructose, and ribose are more sensitive to camptothecin (doses as indicated) than low glucose (mean ± SD **$P < 0.01$ ***$P < 0.001$).

© 2020 The Authors

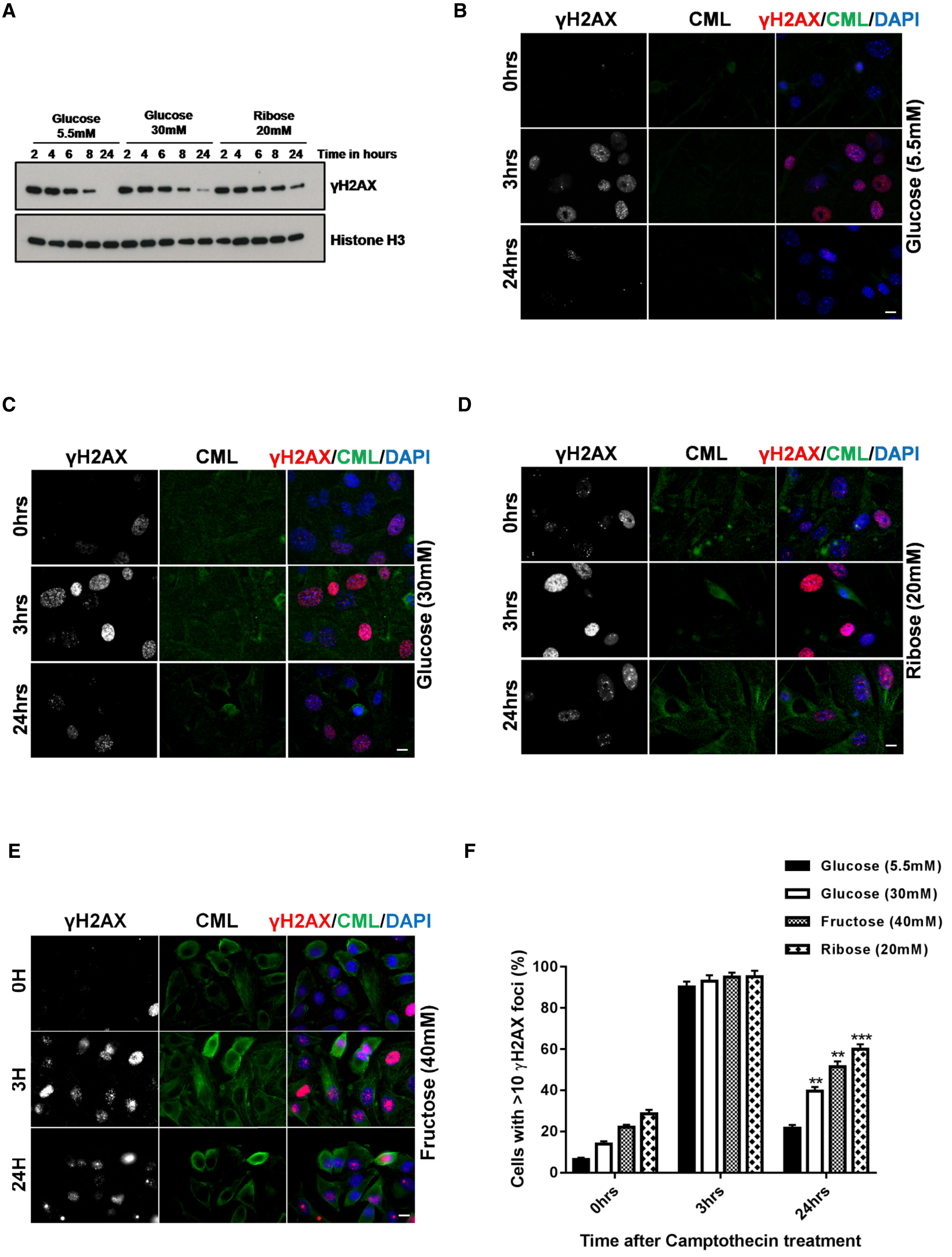

Figure EV3.

 © 2020 The Authors

Figure EV4. Elevated sugar level affects the DNA repair potential of the *db/db* mice model.

A Representative immunoblots of lungs harvested from 4-month-old *+/db* (non-diabetic lean control) or (*db*/*db*) diabetic mice and probed for γH2AX. Histone-H3 was used as a loading control.

B Representative images of lungs from 4-month-old non-diabetic lean controls (*+/db*) and age-matched obese (*db*/*db*) diabetic mice, showing DNA damage foci, as marked by γH2AX (in red). Here, tubulin was used as a morphology marker (shown in green); blue nuclear staining represents DAPI (scale 10 μm). The zoomed portions of each image were shown in thick white lines in the upper left corner.

C Representative immunoblots of kidneys harvested from 4-month *+/db* (non-diabetic control) or (*db*/*db*) diabetic mice and probed for γH2AX. Histone-H3 served as a loading control.

D Representative images of kidneys from 4-month-old non-diabetic lean controls (*+/db*) and age-matched obese (*db*/*db*) diabetic mice, showing DNA damage foci, as marked by γH2AX (in red). Here, tubulin was used as morphology marker (shown in green) and blue nuclear staining represents DAPI (scale 10 μm). The zoomed portions of each image were shown in thick white lines in the upper left corner of each image.

E Representative images of cellular senescence staining in lung and kidney of 4-month-old lean controls (*+/db*) versus obese (*db*/*db*) diabetic mice. Sections stained for cellular senescence-associated β-galactosidase [SA-β-Gal] as described in Methods and visualized by bright field and polarized light; the senescent areas are recognized by its bluish-green staining (scale 40 μm). Eosin (pinkish-red) was used as a morphology stain.

© 2020 The Authors

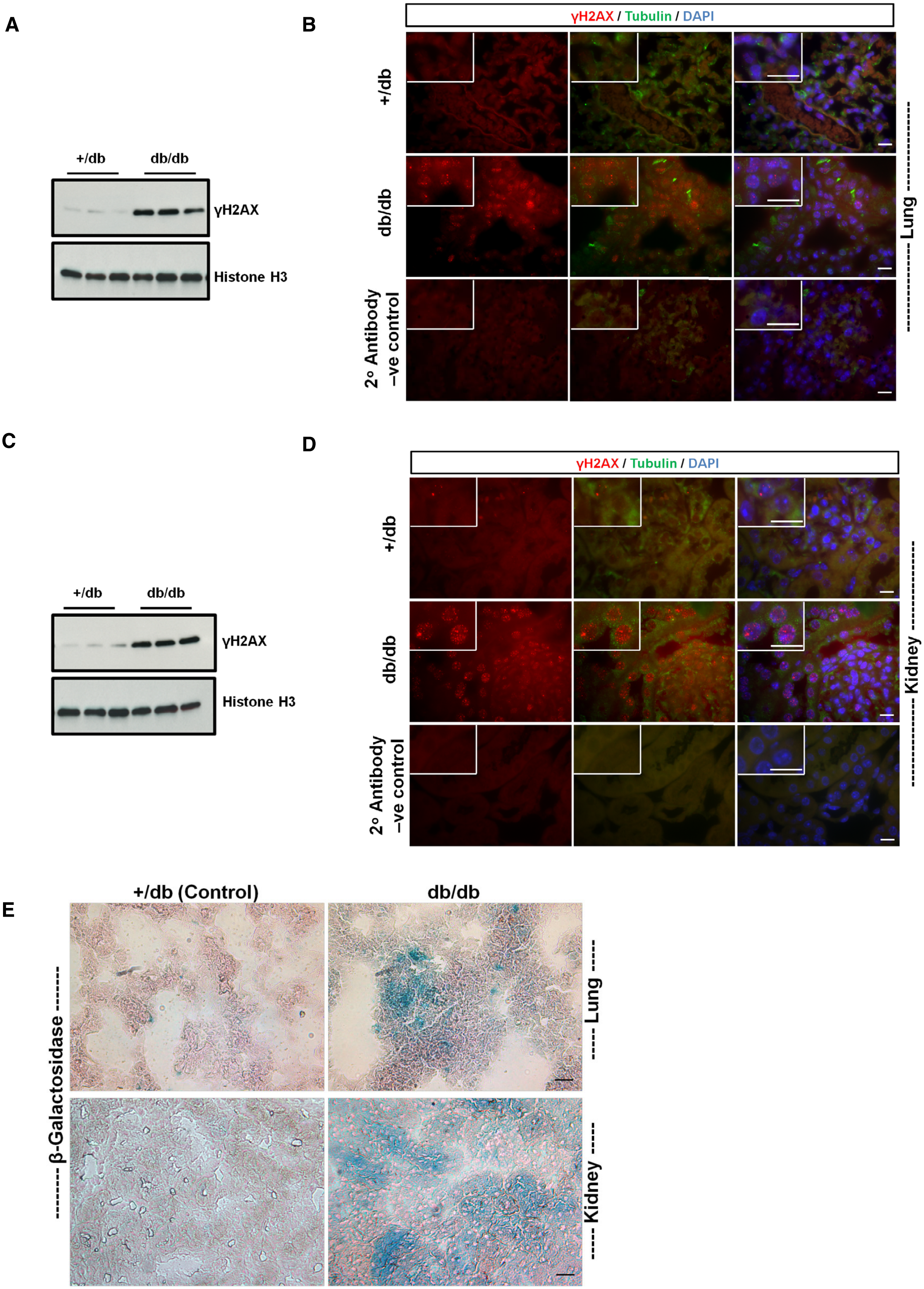

Figure EV4.

© 2020 The Authors

Figure EV5. Molecular characterization of DNA repairs in transduced lungs.

A Representative immunoblots showing the expression of RFP-RAGE, or RFP and γH2AX in transduced lungs of diabetic mice. Data presented in this Figure and in Fig 5 are from the same mice.

B Representative images of lungs from 6-month STZ-diabetic mice transduced with the indicated AAV2/8 virions, as described in Methods. The lungs were harvested 6 weeks after viral transduction and anti-RFP was used for visualizing the expression of virions. Blue nuclear staining represents DAPI.

C Representative images of nuclei positive for the DNA-DSBs marker pATM in lungs from 6-month STZ-diabetic mice and control mice, transduced with the indicated AAV2/8 virions as described in Methods. The lungs were harvested 6 weeks after viral transduction. The empty vector expressing only RFP, served as control. Mean ± SD of six animals per group is shown. Green cytoplasmic staining represents α-tubulin; blue nuclear staining represents DAPI (scale 10 μm). No primary antibody control served as a negative control (shown from RFP group) of the staining.

D Quantitative analysis of pATM-positive nuclei in lungs of 6-month STZ-induced diabetic mice with respective RAGE virions as described in Fig 4 (mean ± SD; $**P < 0.01$, ($N = 8$).

E Quantitative analysis of the static compliance in lungs from 6-month STZ-induced diabetic mice, transduced with AAV2/8 as described in Fig 4 (mean ± SD, $*P < 0.05$, $N = 8$).

F Quantitative analysis of transduction effects of RAGE (AA or EE) or RFP pro-inflammatory, fibrotic, and SASP gene expression in lung tissue. The mRNA of pro-inflammatory, fibrotic, and SASP cytokines was significantly suppressed in RAGE-EE transduction group. The data were normalized against control value (mean ± SD, $*P < 0.05$, $**P < 0.01$).

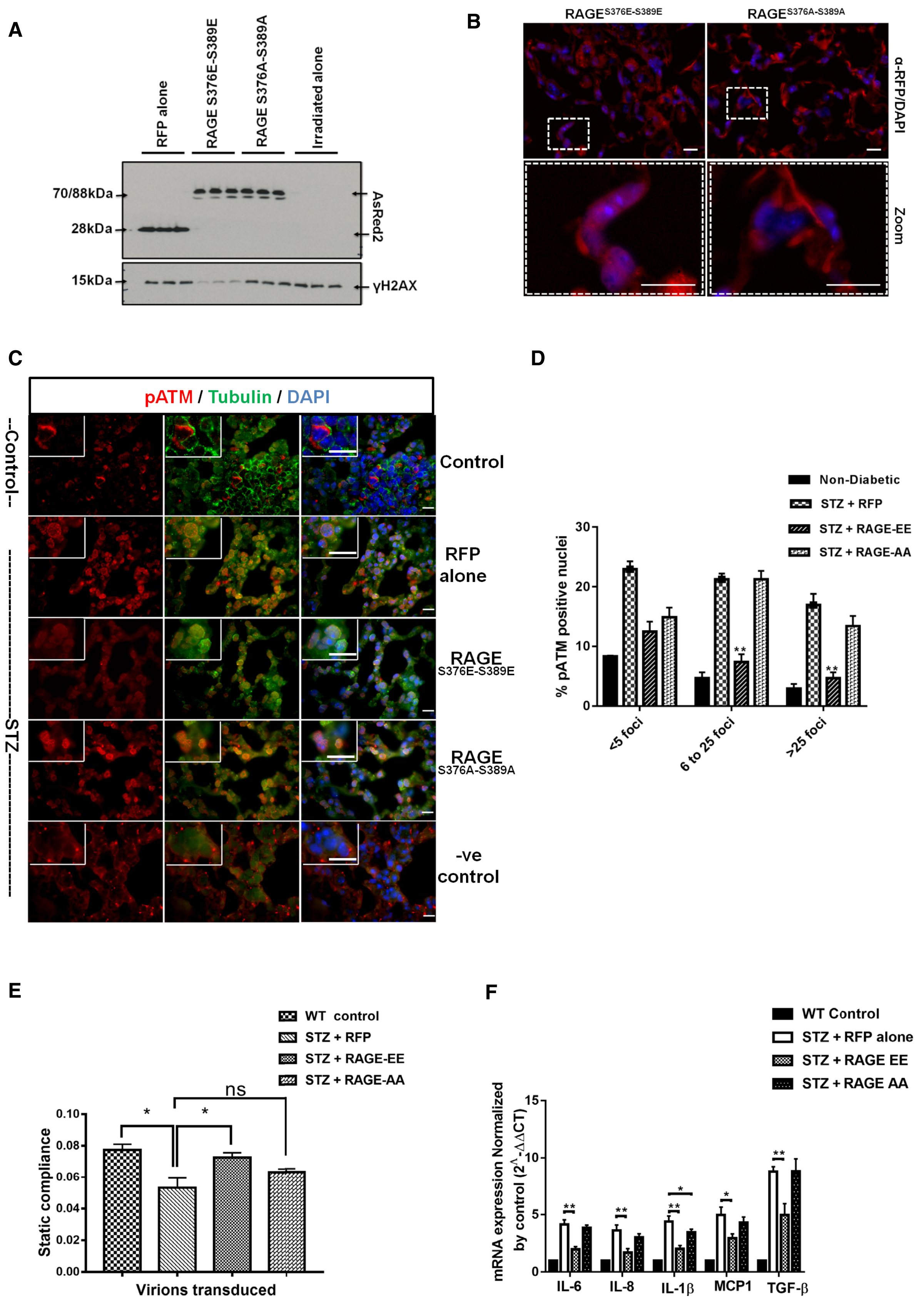

Figure EV5.

© 2020 The Authors
