# Supplementary material for: Compromised DNA repair is responsible for diabetes‐associated fibrosis
Source: EMBO J. 2020 Apr 27;39(11):e103477. doi: 10.15252/embj.2019103477 (PMC7265245; doi:10.15252/embj.2019103477)
Supplement: Supplementary file 3 — Movie EV1 [file EMBJ-39-e103477-s003.zip › Legend_Movie_EV1.rtf]

Movie EV1:  A549 cells showing the live recruitment of hPARP-mCherry (red) and hXRCC4-GFP (green), at the site of laser-induced DNA-DSBs from the cells pre-treated with the indicated reducing sugars (here, LG represents low glucose (5.5mM); HG represents high glucose (30mM) for 5 days. Label Fruct represents fructose (30mM), and label Ribose represents ribose (20mM), for 3 days).
