# Supplementary figures and images for: Compromised DNA repair is responsible for diabetes‐associated fibrosis
Source: EMBO J. 2020 Apr 27;39(11):e103477. doi: 10.15252/embj.2019103477 (PMC7265245; doi:10.15252/embj.2019103477)

Uncropped Immunoblot for figure EV1A (Older number was EV2A)

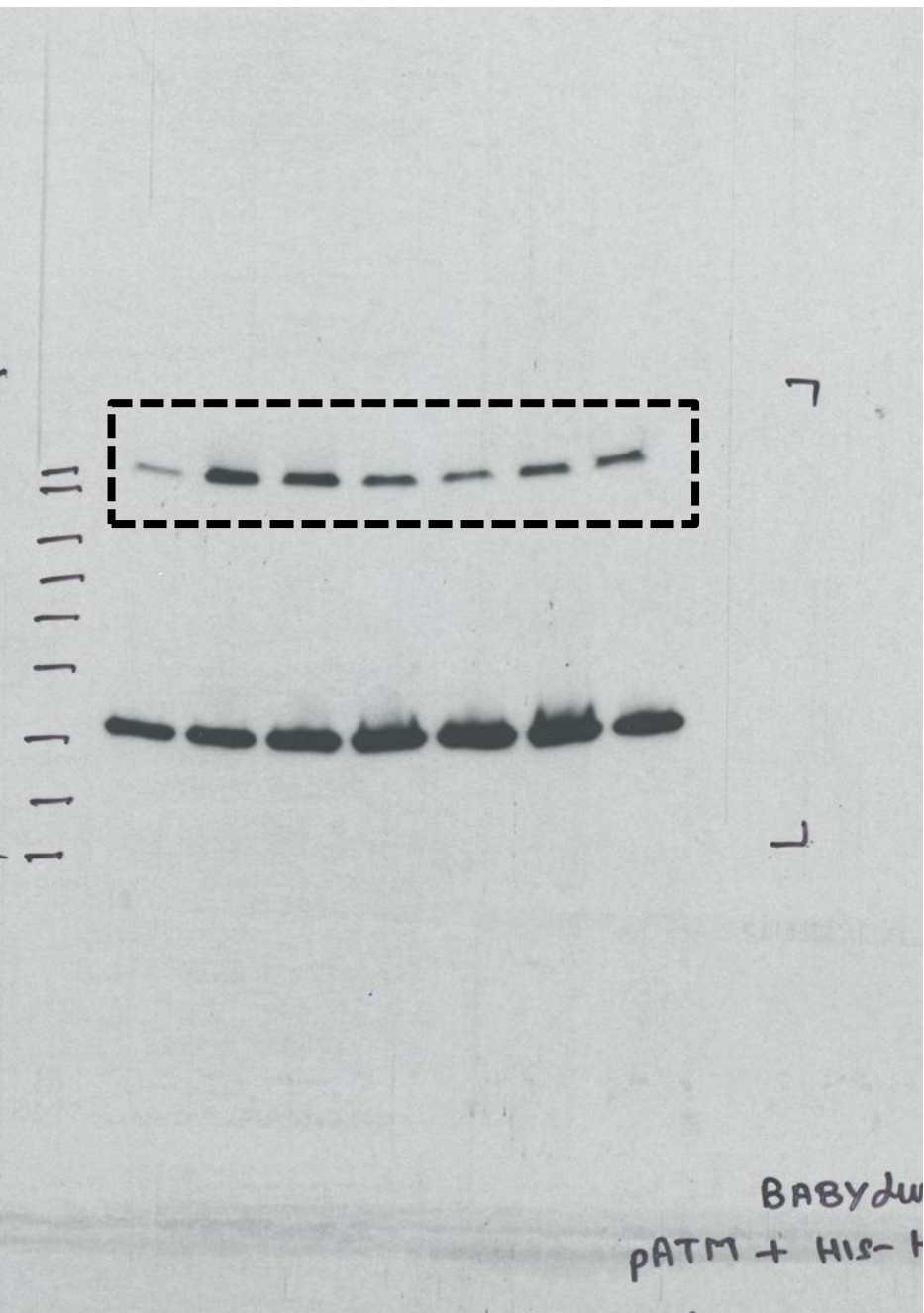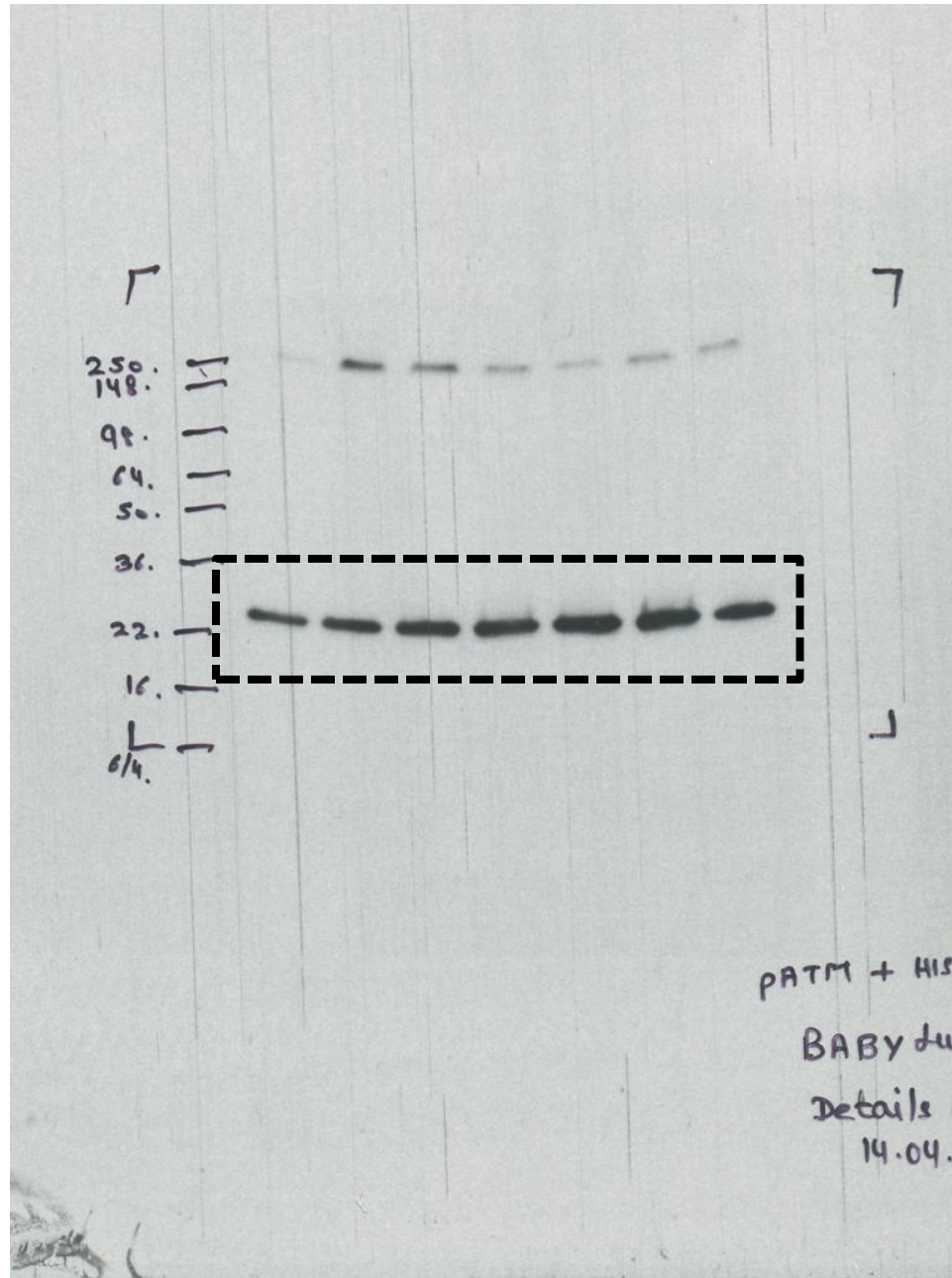

Supplement: Supplementary file 4 — Source Data for Expanded View and Appendix [file EMBJ-39-e103477-s004.zip › EMBOJ-2019-103477R1-Figure_EV1_Source_Data.pdf]
