# Supplementary material for: Compromised DNA repair is responsible for diabetes‐associated fibrosis
Source: EMBO J. 2020 Apr 27;39(11):e103477. doi: 10.15252/embj.2019103477 (PMC7265245; doi:10.15252/embj.2019103477)

Uncropped Immunoblot for Appendix figure S4C (Older number was EV7C)

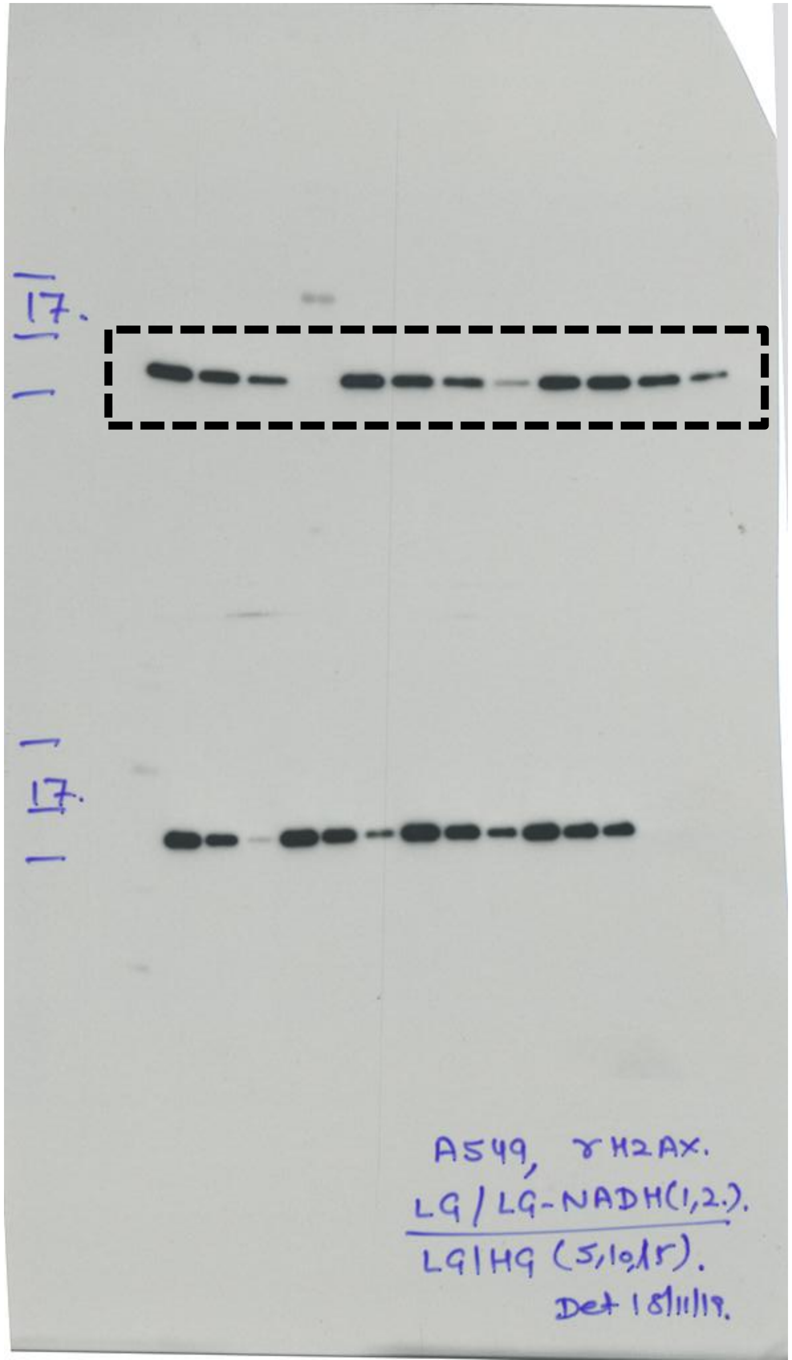

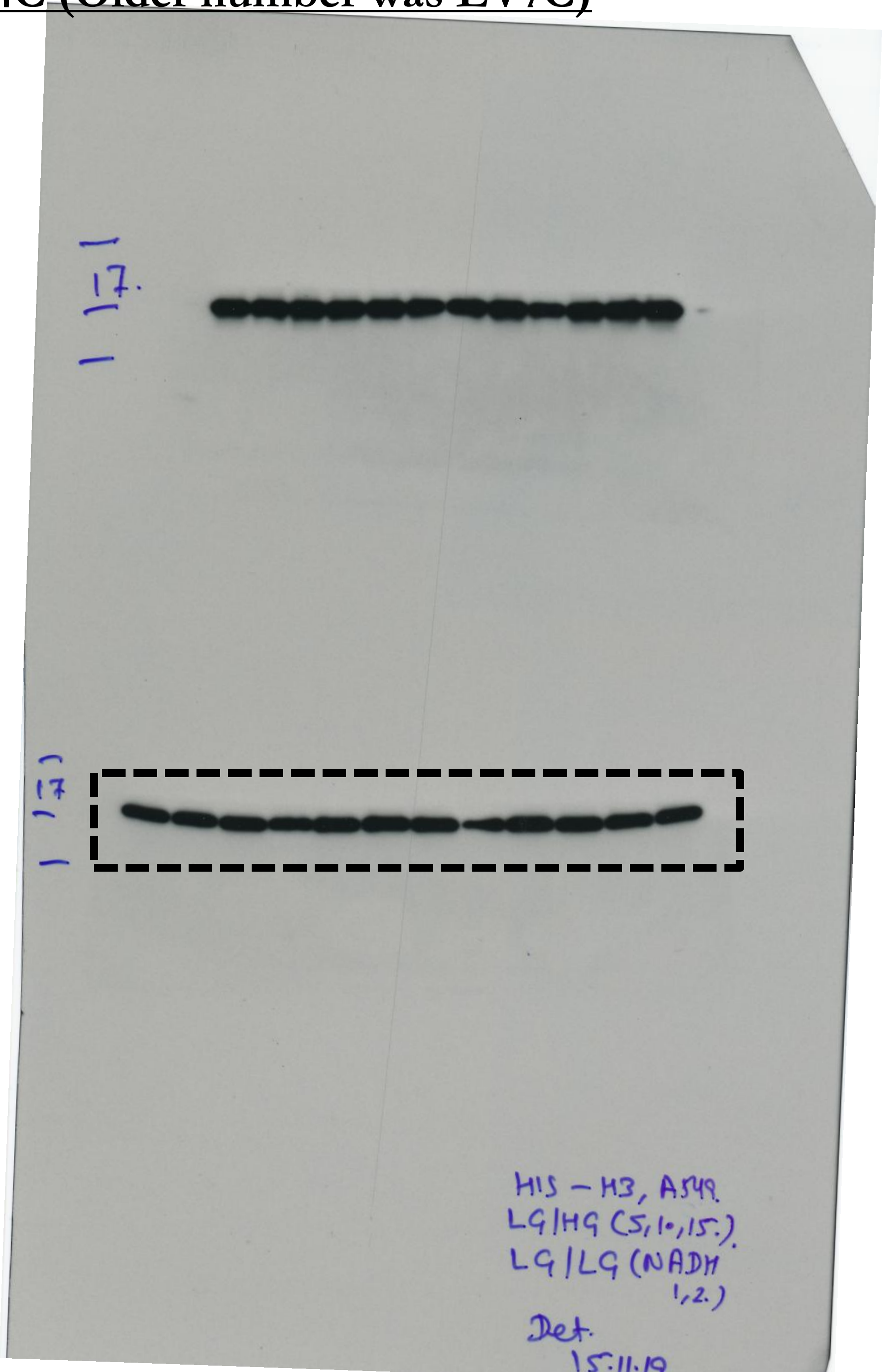

Uncropped Immunoblot for Appendix figure S4D (Older number was EV7D)

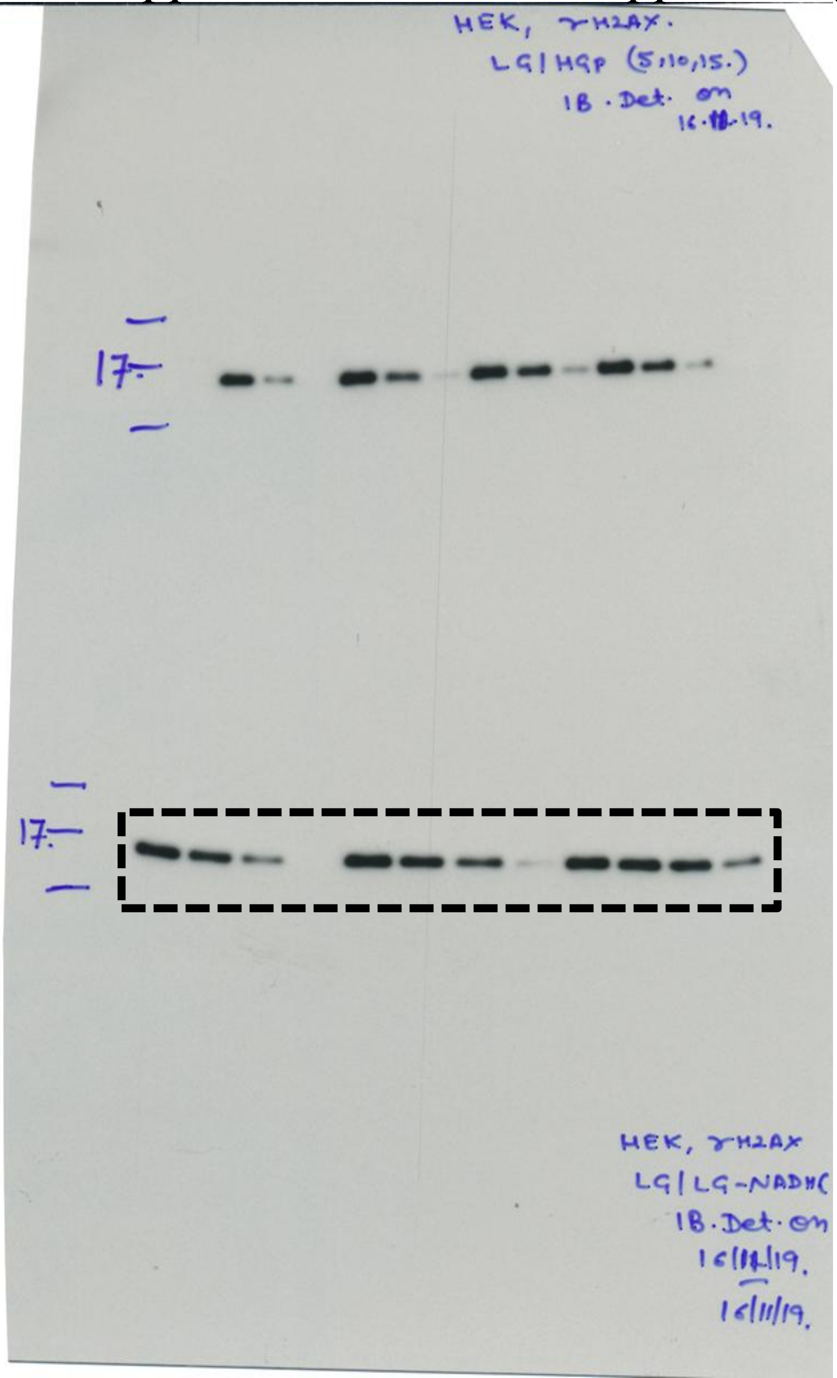

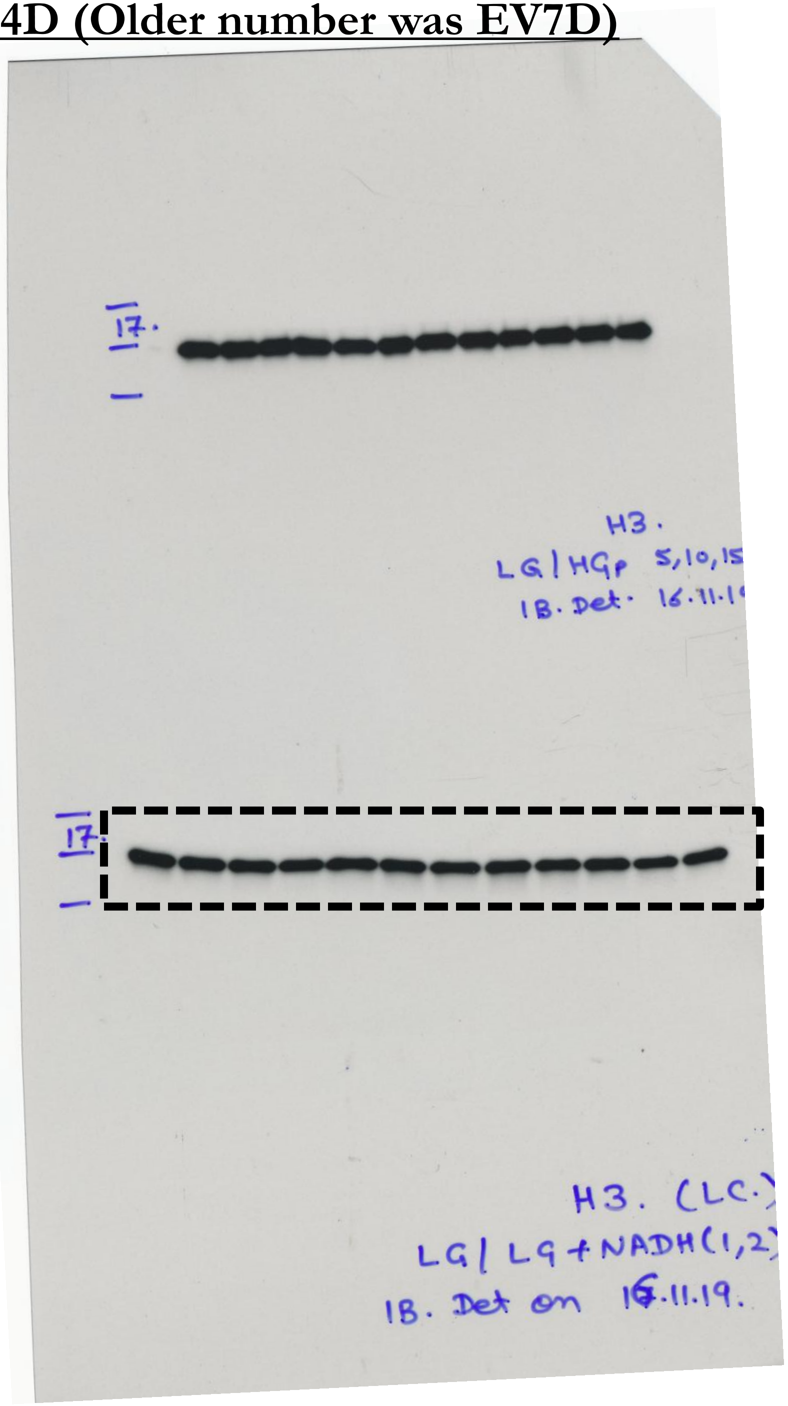

Supplement: Supplementary file 4 — Source Data for Expanded View and Appendix [file EMBJ-39-e103477-s004.zip › EMBOJ-2019-103477R1-Apendix_Figure_Source_Data.zip!/EMBOJ-2019-103477R1-Figure_S4_Source_Data.pdf]
